# Calpain-3 Impairs Cell Proliferation and Stimulates Oxidative Stress-Mediated Cell Death in Melanoma Cells

**DOI:** 10.1371/journal.pone.0117258

**Published:** 2015-02-06

**Authors:** Daniele Moretti, Barbara Del Bello, Giulia Allavena, Alessandro Corti, Cinzia Signorini, Emilia Maellaro

**Affiliations:** 1 Department of Molecular and Developmental Medicine, University of Siena, Siena, Italy; 2 Department of Translational Research and New Technologies in Medicine and Surgery, Medical School, University of Pisa, Pisa, Italy; 3 Istituto Toscano Tumori (ITT), Firenze, Italy; North Carolina State University, UNITED STATES

## Abstract

Calpain-3 is an intracellular cysteine protease, belonging to Calpain superfamily and predominantly expressed in skeletal muscle. In human melanoma cell lines and biopsies, we previously identified two novel splicing variants (hMp78 and hMp84) of Calpain-3 gene (*CAPN3*), which have a significant lower expression in vertical growth phase melanomas and, even lower, in metastases, compared to benign nevi. In the present study, in order to investigate the pathophysiological role played by the longer Calpain-3 variant, hMp84, in melanoma cells, we over-expressed it in A375 and HT-144 cells. In A375 cells, the enforced expression of hMp84 induces p53 stabilization, and modulates the expression of a few p53- and oxidative stress-related genes. Consistently, hMp84 increases the intracellular production of ROS (Reactive Oxygen Species), which lead to oxidative modification of phospholipids (formation of F_2_-isoprostanes) and DNA damage. Such events culminate in an adverse cell fate, as indicated by the decrease of cell proliferation and by cell death. To a different extent, either the antioxidant N-acetyl-cysteine or the p53 inhibitor, Pifithrin-α, recover cell viability and decrease ROS formation. Similarly to A375 cells, hMp84 over-expression causes inhibition of cell proliferation, cell death, and increase of both ROS levels and F_2_-isoprostanes also in HT-144 cells. However, in these cells no p53 accumulation occurs. In both cell lines, no significant change of cell proliferation and cell damage is observed in cells over-expressing the mutant hMp84^C42S^ devoid of its enzymatic activity, suggesting that the catalytic activity of hMp84 is required for its detrimental effects. Since a more aggressive phenotype is expected to benefit from down-regulation of mechanisms impairing cell growth and survival, we envisage that Calpain-3 down-regulation can be regarded as a novel mechanism contributing to melanoma progression.

## Introduction

Calpains (Clan CA-C2, EC 3.4.22.17) constitute a superfamily of Ca^2+^-regulated, intracellular cysteine proteases, evolutionarily well-conserved from bacteria to mammals. On the basis of their domain structure, a complex classification of calpains and calpain homologs, divided into “classical” and “non-classical”, has been recently proposed [1]. In humans, 9 out of the 15 calpain genes code for classical calpains, with alternative splicing variants also generated. On the basis of expression profile, 6 out of the 15 mammalian genes are considered to be tissue-specific, and defects of the corresponding calpains have been associated with tissue-specific pathological phenotypes. Among these calpains, particular emphasis has been placed on *CAPN3* gene product, Calpain-3 (or p94), predominantly expressed in skeletal muscle. It proves to be crucial for muscle cell homeostasis, as demonstrated in Limb-Girdle Muscular Dystrophy type 2A (LGMD2A, or calpainopathy), which is characterized by different *CAPN3* point mutations and by muscle hypotrophy, hypoplasia and myonuclear apoptosis [2,3].

In melanoma cell lines and in melanoma biopsies, we have previously identified two novel splicing variants of *CAPN3*, namely hMp78 and hMp84 (GenBank accession number: EU91850 and EU91851, respectively) [4]. We demonstrated that both proteins are localized in cytoplasm, and the autolytic cleavage form of the longer variant is also localized in nucleoli. Interestingly, among melanocytic lesions, the expression of these variants is significantly lower, compared to benign nevi, in the most aggressive lesions, i.e. in vertical growth phase melanomas and, even lower, in metastases. Moreover, in cisplatin-treated melanoma cells, an increased (auto)proteolytic activation of hMp84 variant occurs [4]. This event, in analogy with the drug-induced activation of conventional calpains [5], suggested an active role of this isoform in the apoptotic machinery of melanoma cells.

A few papers from other Authors have pointed out a potential role of Calpain-3 in human melanoma tumorigenesis and progression, as indicated by the higher expression levels of *CAPN3* in melanoma tissues compared to other tumor types [6], and by *CAPN3* down-regulation in melanoma cells sensitive to interferon-γ [7] or undergoing drug-induced terminal differentiation [8]. More recently, Calpain-3 down-regulation has been also demonstrated in the acquisition of a highly invasive metastatic phenotype [9]. Moreover, in an interesting study of veterinary oncology, Calpain-3 has been shown to be activated in urothelial tumors of cattle [10].

Against this background, in the present study we over-expressed the longer *CAPN3* variant (hMp84) in A375 and HT-144 melanoma cells, in order to better understand the pathophysiological role played by Calpain-3 in melanoma cells, and the underlying biochemical and molecular mechanisms regulated by this calpain. Our results demonstrate that over-expression of hMp84 impairs cell proliferation and, concomitantly, induces cell death. As a mechanism responsible for cell damage, a redox imbalance, due to increased production of Reactive Oxygen Species (ROS), is shown to play a major role.

## Materials and Methods

### Cell culture and treatments

Human melanoma A375 and HT-144 cells (from ATCC, cat. n. CRL-1619 and HTB-63, respectively) (American Type Culture Collection, Manassas, VA) were cultured in Dulbecco’s modified Eagle’s medium (DMEM) with 4.5 g/L glucose (Sigma-Aldrich, St. Louis, MO) and in RPMI-1640 medium (Sigma), respectively, containing 10% heat-inactivated foetal bovine serum (Invitrogen Life Technologies, Carlsbad, CA), 50 mg/L gentamycin, and 2 mM L-glutamine, in a 37°C incubator, under 95% air and 5% CO_2_. For routine reseeding and for experiments, cells were *harvested with* PBS-EDTA 1 mM, pH 7.4. In selected experiments, cells over-expressing the hMp84 variant of Calpain 3 and control cells transfected with empty vector (produced as detailed below) were treated in fresh medium with 1 μM Pifithrin-α (PFT) (Sigma-Aldrich) or 5 mM *N*-Acetyl-L-Cysteine (NAC) (Sigma-Aldrich). These compounds have been preliminarily tested, in order to choose the lowest effective concentrations giving no or minor toxicity in control cells.

At different experimental times, we harvested and analyzed the population of still adhering cells together with or separately from the population of floating cells, depending on the investigations to be performed, as specified in Results and Figure Legends. Cell proliferation was evaluated as total cells (adhering *plus* floating), counted in a Bürker chamber. The percentage of floating on total cells was used as a first quantitative indication of cell damage.

### hMp84 cloning, site-directed mutagenesis, and transient transfection

The human *CAPN3* gene (hMp84 variant) was cloned from the human melanoma cell line HT-144, previously characterized by us [4]. Total RNA was extracted by using RNeasy Mini Kit (Qiagen, Valencia, CA) according to manufacturer’s instructions. cDNA was obtained from 1 μg of total RNA by using High Capacity cDNA Reverse Transcription Kit and Oligo dTs as primers (Invitrogen Life Technologies). hMp84 was amplified with specific primers (S1 Table) and cloned into pcDNA3.1(+) plasmid in BamHI-XhoI, by using *E*. *coli* (DH5α) as host. Positive clones were sequenced to verify the absence of mutations. In order to mutate hMp84 in the active site, Quickchange II XL site-directed mutagenesis kit (Agilent Technologies, Santa Clara, CA) was used, according to manufacturer’s instructions. Specific primers (S1 Table) were designed to replace cysteine (at position 42) with serine. pcDNA3.1(+)-hMp84 was used as template. The resulting vector (pcDNA3.1(+)-hMp84^C42S^) was then sequenced to verify the correct mutagenesis. DNA for transfection experiments was prepared using Qiafilter Plasmid Maxi Kit (Qiagen), according to manufacturer’s instructions, in *E*. *coli* (DH5α) as host. The resulting vector (containing wild or mutated hMp84) was used to transfect melanoma cells, by using Attractene Trasfection Reagent (Qiagen). Cells, seeded the day before, were incubated with the transfectant mixture for 6 hours, then the medium was changed; the same procedure was used for control cells, where the empty vector was employed.

To determine transfection efficiency, plasmids pEGFP-N2 (Clontech, Mountain View, CA) and pEGFP-N2-hMp84, both containing the reporter gene for Enhanced Green Fluorescent Protein (EGFP), were used. EGFP-expressing cells (at least 200 cells scored in each experiment) were directly visualized by fluorescence microscopy (Nikon Eclipse Ti, Japan). The average transfection efficiency was 40% and 30%, for A375 and HT-144 cells, respectively.

### LDH release assay

The release of intracellular lactate dehydrogenase (LDH) in the culture medium was evaluated by using LDH-P Kit (Sclavo Diagnostics International, Siena, Italy), according to manufacturer’s instructions, with minor modifications. An equal number of cells was seeded for all samples in each experiment, and the *ratio* of seeded cells/medium volume was kept constant among different experiments. Forty μl of cell-free culture medium was collected for each sample and added to 160 μl of the assay mixture. LDH activity was measured at room temperature in a microplate reader (Victor3 Multilabel Counter, Perkin-Elmer, Waltham, MA), absorbance decrease was recorded at 340 nm over a period of 0.5–8 min, and results were expressed as ∆Abs/min/10^-3^.

### Nuclear morphology analysis

Adhering and floating cells were collectively harvested and fixed at room temperature in 4% paraformaldhehyde for 30 min. After a gentle centrifugation, fixed cells were stained with the chromatin dye Hoechst 33342 (2 μM in RPMI without serum) (Sigma-Aldrich) for 30 min at room temperature, and cell suspension was placed on a slide, with a coverslip over cells. The nuclear morphology was observed under a fluorescence microscope (Nikon, Tokyo, Japan) equipped with a DAPI filter. Images were captured with a CCD camera and imported into NISElements Imaging software. At least 300 cells were scored for each sample in each experiment. Apoptotic cells were detected as nuclei with condensed chromatin (brighter fluorescence), and as smaller or fragmented nuclei.

### Fluorimetric assay for caspase-3/-7 activity

Caspase-3/-7 enzymatic activity was measured essentially as previously described [11]. Briefly, the harvested (adhering *plus* floating) cells, after washing with PBS, were resuspended in ice-cold lysis buffer (20 mM Hepes-NaOH, pH 7.5, containing 10% sucrose, 0.1% CHAPS, 0.2% NP-40, 1 mM EDTA, 5 mM DTT, 1 mM PMSF, and protease inhibitor cocktail (Sigma-Aldrich)), and sonicated for 10 s (Vibracell Sonicator; amplitude 60, 25 W). After centrifugation, the supernatant of cell lysates (100 μg/ml of protein) were incubated at 25°C with the substrate ac-Asp-Glu-Val-Asp-7-amido-4-methylcoumarin (ac-DEVD-AMC, 40 μM) (Alexis Enzo Life Sciences, Farmingdale, NY) in the assay buffer (lysis buffer, where Hepes and NP-40 were 100 mM and 0.1%, respectively). Cleavage of the fluorogenic substrate was monitored by AMC release, in the microplate reader (excitation/emission wavelengths: 380/460 nm). Caspase-3/-7 activity, determined in the linear portion of the progress curve, was expressed as Arbitrary Units of Fluorescence (ΔAUFs)/min/mg protein.

### Single-cell gel electrophoresis (Comet assay)

DNA damage was measured using the alkaline Comet assay, as described by Corti et al. [12]. At 24 h after transfection, harvested cells were suspended in 0.6% low melting point agarose. 2 x 10^4^ cells were dispensed onto glass microscope slides previously coated with 1% normal melting point agarose, and the slides were left overnight in ice-cold lysis buffer (100 mM EDTA, 2.5 M NaCl, 10 mM Tris base, pH 10, containing 1% Triton X-100 (v/v) freshly added). After washing with distilled water, slides were placed for 20 min in a horizontal electrophoresis tank, containing ice-cold alkaline electrophoresis solution (300 mM NaOH, 1 mM EDTA), to allow DNA unwinding. Electrophoresis was conducted for 20 min (30 V, 300 mA) at 4°C. Slides were neutralised with 0.4 M Tris—HCl, pH 7.5 for 20 min, washed with distilled water, and then allowed to dry. All procedures were carried out under subdued light to minimise background DNA damage. For staining, slides were re-hydrated in distilled water, incubated for 20 min with a freshly made solution of 2.5 μg/ml propidium iodide, washed again and allowed to dry. Comets were visualised with a fluorescence microscope (Leica, Wetzlar, Germany) at 200× magnification, and a total of 100 cells *per* sample (50 *per* duplicate slide) were analyzed. Images were captured by an on-line Leica DFC320 camera, and the percentage of DNA in the comet tail (% tail DNA), which quantifies the DNA damage, was calculated for each cell by the CometScore software (TriTek Corporation, Sumerduck, VA).

### Total F_2_-isoprostanes determination

The levels of total (free *plus* esterified) F_2_-isoprostanes (F_2_-IsoPs) in the cell suspensions were determined by gas chromatography/negative ion chemical ionization tandem mass spectrometry (GC/NICIMS/MS). After 24 h of transfection, adhering cells were harvested with PBS-EDTA 1 mM, pH 7.4 and the pellet was resuspended with the antioxidant butylated hydroxytoluene (BHT) (final concentration 90 μM); subsequently, the samples were sonicated for 30 seconds and then incubated at 45°C for 45 min in presence of 0.5 N KOH for the basic hydrolysis. The samples were then acidified to pH 3 with HCl (final concentration 0.5 N), and spiked with tetradeuterated PGF_2_α (500 pg) as internal standard. After extraction with ethyl acetate, the total lipids were applied to an aminopropyl (NH_2_) cartridge, and the final eluates were derivatized to pentafluorobenzyl ester and trimethylsylil ethers and examined by GC/ NICI-MS/MS as previously reported [13]. Total F_2_-IsoPs levels were normalized for cell protein content.

### Measurement of ROS generation in intact cells

To assess the production of intra- or extra-cellular Reactive Oxygen Species (ROS) in intact cells, two different fluorimetric assays were used, both performed in still adhering, viable cells, in order to exclude any ROS formation as secondary event following cell death. Intracellular production of ROS was measured by oxidation of 2’-7’-dichloro-fluorescin diacetate (H_2_DCF-DA) to 2’-7’-dichloro-fluorescein (DCF). H_2_DCF-DA readily diffuses into cells, where it is de-esterified into a non-fluorescent derivative and trapped within cells; when oxidated (by H_2_O_2_ and other oxidant species), it turns into the highly fluorescent DCF. Briefly, 3x10^4^ cells were seeded in 24-well plates, transfected, and left untreated or treated with NAC or PFT-α. At different time points, cells were washed with Krebs buffer containing 0.5 mM CaCl_2_ (KBC), incubated for 30 min at 37°C in KBC containing 5 μM H_2_DCF-DA (Molecular Probes, Eugene, OR), and then washed with KBC. Fluorescence was measured in the microplate reader (excitation/emission wavelengths: 490/530 nm) for 60 min. Extracellular production of ROS was measured using the horseradish peroxidase-linked Amplex Red (A6550), which reacts with H_2_O_2_ to produce the fluorescent oxidation product, resorufin. Briefly, cells, prepared as above, were incubated for 1 hour with 50 μM A6550 and 0.1 U/ml horseradish peroxidase type II, with or without the inhibitor of NADPH oxidase, diphenylen iodonium chloride (DPI, 10 μM) (Sigma-Aldrich). Fluorescence was subsequently measured in the microplate reader (excitation/emission wavelenghts: 560/590 nm) for 90 min. ROS production was expressed as Arbitrary Units of Fluorescence (ΔAUF)/min/cell, where the actual cell number was evaluated in each well at the end of the assays. A blank, run in KBC with all reagents and without cells, was subtracted to all samples.

### Western blot analysis

Cell lysates, as prepared for caspase activity assay, were subjected to SDS-PAGE and transferred to nitrocellulose membranes (AppliChem GmbH, Darmstadt, Germany). The membranes, blocked in 10% non-fat milk in PBS-Tween 0.05% (PBST), were probed overnight at 4°C with primary antibodies diluted in PBST-1% non-fat milk: mouse monoclonal anti-calpain-3 (dilution 1:50) (CALP12A2, Novocastra, Newcastle upon Tyne, UK), mouse monoclonal anti-p53 (dilution 1:1000) (SC-126, Santa Cruz Biotechnology, Santa Cruz, CA), and rabbit polyclonal anti-PARP1/2 (dilution 1:1000) (SC-7150, Santa Cruz Biotechnology,). After incubation with horseradish peroxidase-conjugated secondary antibodies (Sigma-Aldrich) at room temperature for 1 h, protein bands were visualized by an enhanced chemiluminescence reaction system (Super Signal West Pico, Thermo Scientific, Rockford, IL). Membranes were then developed on Hyperfilm ECL (Amersham GE Healthcare, UK) and protein bands were quantified by densitometric analysis (image processing software ImageJ). Before adding primary antibodies, equal gel loading, quality control, and transfer efficiency among samples were assessed by reversible Ponceau S protein staining on membranes. Equal loading was also assessed by mouse monoclonal anti-β-actin-HRP conjugated antibody (dilution 1:50,000) (Sigma-Aldrich).

### PCR Array

Gene expression analysis in transfected cells was carried out by using RT^2^ Profiler PCR Array: Human Oxidative Stress (PAHS-065Z, Qiagen), following manufacturer’s instructions. Briefly, total RNA was extracted with RNeasy Mini Kit (Qiagen), and genomic DNA was removed by using in-column DNA digestion (Qiagen). Total RNA (1 μg) was retro-transcribed by using RT^2^ First Strand Kit (Qiagen), with Oligo dTs as primers. The obtained cDNA was used for Real-Time PCR detection with RT^2^ SYBR Green/ROX qPCR MasterMix (Qiagen). Amplification protocol was set up according to manufacturer’s instructions for Applied Biosystems StepOnePlus instrument (Applied Biosystems, Foster City, CA). Data were analyzed by using Web-based PCR Array Data Analysis Software at: http://sabiosciences.com/pcrarraydataanalysis.php.

### Real-Time PCR analysis

RNA was extracted as above described. cDNA was obtained from 1 μg of total RNA using High Capacity cDNA Reverse Transcription Kit (Invitrogen Life Technologies) and random primers. Gene expression level was measured in triplicates by Real-Time PCR, using StepOnePlus (Applied Biosystems) and Power SYBR Green PCR Master Mix (Invitrogen Life Technologies), and normalized to the endogenous reference gene β-Actin (S1 Table for the primers used). The thermocycling conditions were: 10 minutes at 95°C, followed by 40 cycles of denaturation (95°C for 15 s) and 60°C for 1 minute, for annealing and extension. Melting curve analysis was used to confirm specificity of the obtained results. Gene expression values in hMp84-transfected cells were expressed as fold-change over empty vector-transfected cells, using 2^-∆∆c(t)^ parameter [14].

### Statistics

Results are presented as mean ± SE, and the statistical significance of the differences among the groups was determined by the Student’s *t*-test. Where stated, results are presented as mean ratio (over EV-cells) ± 95% confidence interval, and the statistical significance was determined by means of 95% confidence interval.

## Results

### Calpain-3 over-expression in A375 melanoma cells

**Fig 1:**
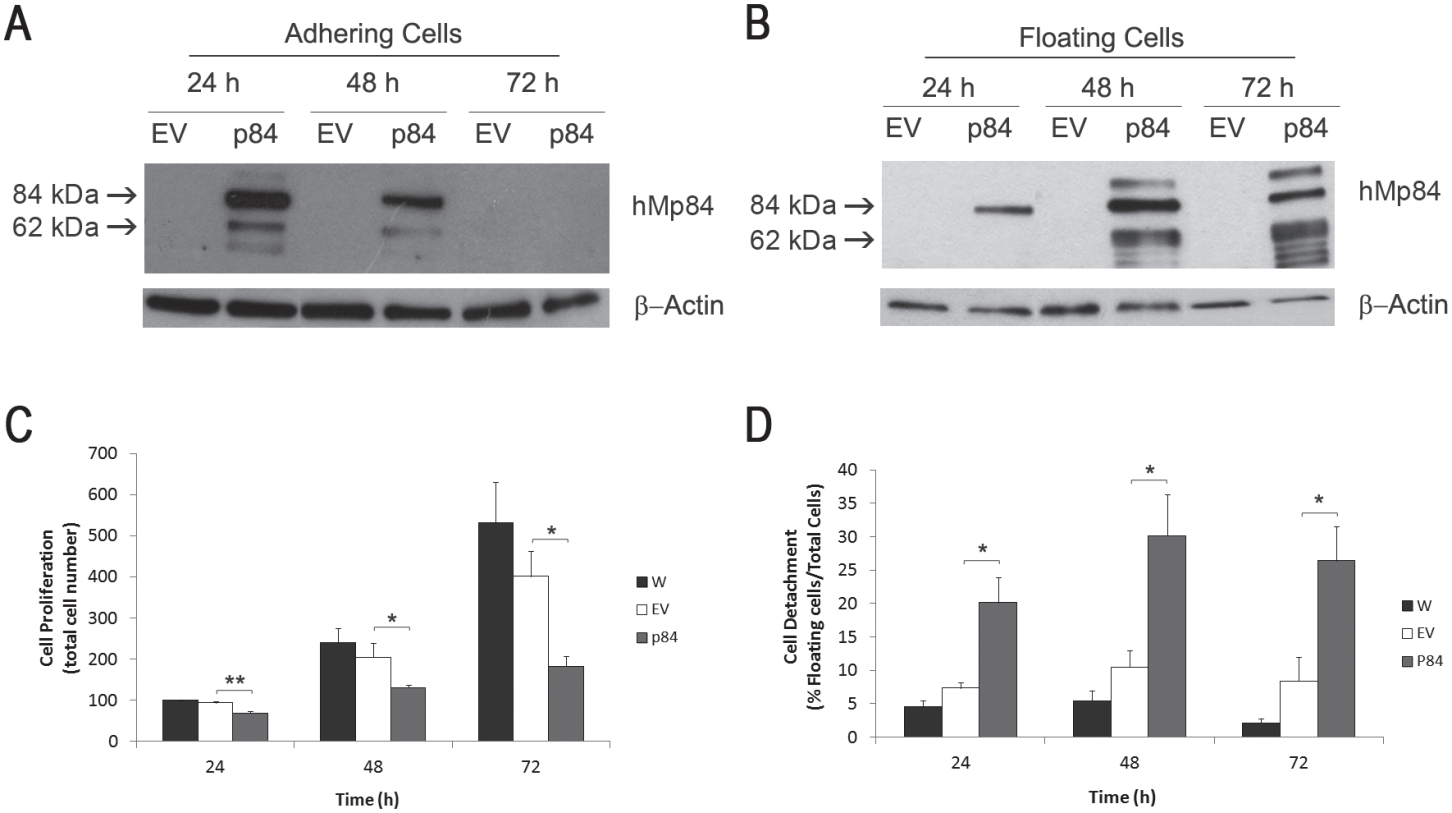
Transient over-expression of Calpain-3 (variant hMp84) impairs cell proliferation and leads to cell detachment in A375 melanoma cells. At 24, 48 and 72 h after transfection, hMp84 protein (p84) was evaluated by Western blot in empty vector- (EV) and in hMp84-transfected cells (p84), both in still adhering, viable cells (**A**) and in floating cells (**B**), separately harvested. A typical Western blot out of three is shown. At the same times after transfection, cell proliferation (total cell number) (**C**) and cell detachment (% of floating cells on total cells) (**D**) were evaluated in parental, non-transfected cells (W), in EV-cells and in p84-cells. Results are presented as mean ± S.E. of at least four independent experiments. *P<0.05; **P<0.01.

In order to over-express the Calpain-3 variant hMp84 (hereafter called p84) in human melanoma cells, preliminary experiments performed in a few cell lines were aimed to produce stably-transfected cells, through selection of neomycin-resistant clones; unfortunately, we failed to obtain stable p84 over-expressing cells, since they all died after 7–10 days of selection. The observation that melanoma cells do not tolerate long-lasting, high levels of such Calpain-3 variant was a starting and useful indication about the detrimental effect of this protease to melanoma cells, and prompted us to perform experiments in transiently over-expressing cells. For the majority of these experiments, A375 melanoma cells were chosen, due to their satisfactory transfection efficiency (about 40%). After transfection, the expression level of p84 protein was evaluated at different time points. As shown in Fig. 1A and B, in empty vector-transfected cells (hereafter called EV-cells) the full-length p84 is hardly detectable, while in p84 over-expressing cells (hereafter called p84-cells) there is a significant amount of the full-length protein, along with a few additional bands, including a major one of approximately 60 kDa. As we have previously shown by means of a different antibody [4] and similarly to what happens in muscle Calpain-3 [15], these additional bands derive from p84 (auto)proteolysis. In adhering cells, the full-length p84 protein reaches a peak at 24 h, decreasing at 48 h and disappearing at 72 h after transfection. As expected, a huge increase of hMp84 mRNA is also observed at 24 h after transfection in p84-cells (RT-PCR data not shown), compared to non-transfected cells and EV-cells.

### Calpain-3 over-expression inhibits cell proliferation and induces cell death in A375 cells

Compared to EV-cells, p84-cells show a significant, progressive decrease of cell proliferation, evaluated as total cell number (Fig. 1C). Concomitantly, a notable time-dependent increase of floating cells detached from the monolayer is observed, as a first indication of an ongoing cell damage and eventual cell demise (Fig. 1D). Interestingly, p84 expression, which is lost in still adhering cells at 72 h, is enduring at this time in floating, detached cells (Fig. 1B), indicating that the longer lasting p84 expression is just associated to cell damage.

**Fig 2:**
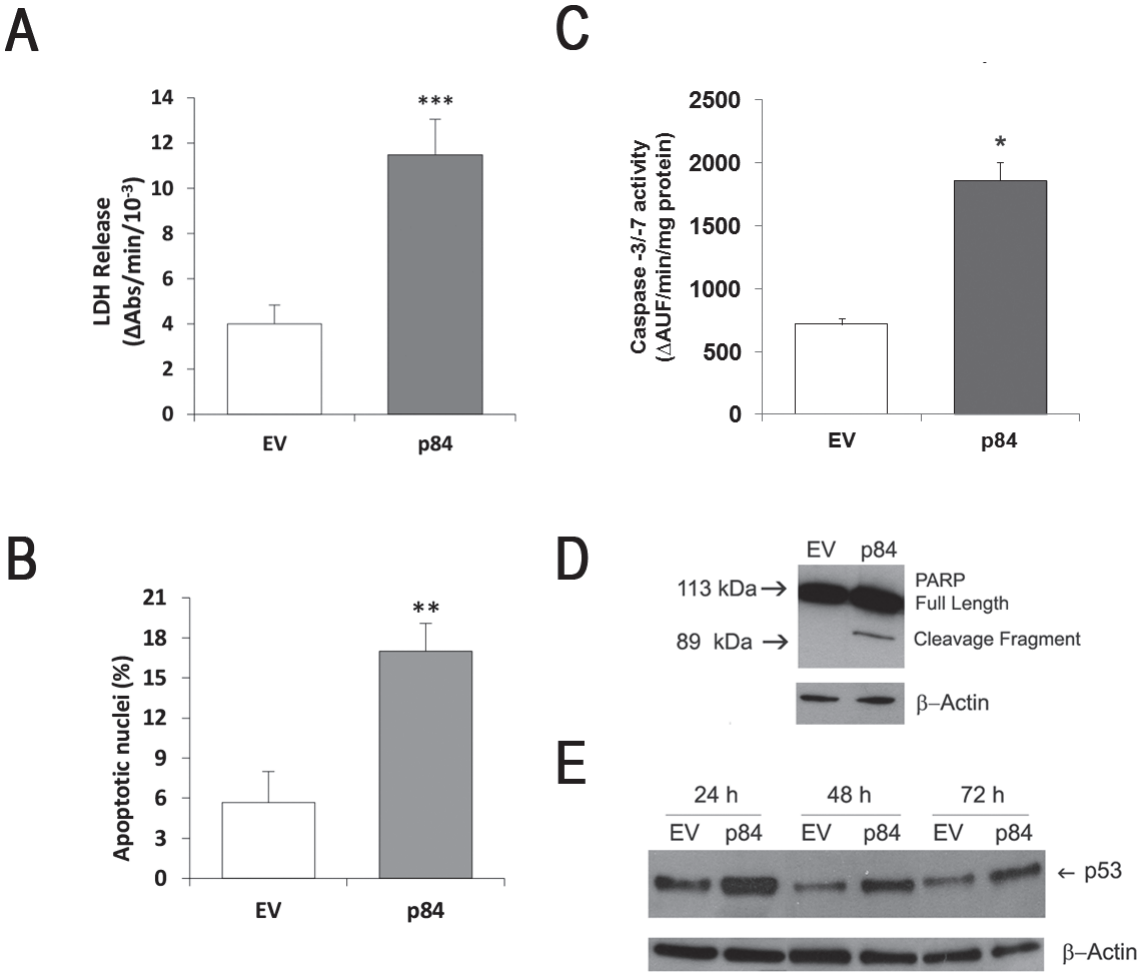
hMp84 stimulates cell death and stabilizes p53 protein in A375 melanoma cells. At 24 h after transfection, cell death was evaluated in EV-cells and in p84-cells as amount of intracellular LDH released in the culture medium (**A**), as percentage of nuclei showing an apoptotic morphology (Hoechst 33258 staining) (**B**), as caspase-3/-7 activation (enzymatic activity) (**C**), and as caspase-mediated proteolysis of poly(ADP-ribose) polymerase (PARP) (a typical Western blot experiment out of three is shown) (**D**). Results are presented as mean ± S.E. of three to six independent experiments. *P<0.05 and ***P<0.001, compared to control EV-cells. At 24, 48 and 72 h after transfection, p53 protein was evaluated by Western blot (**E**), in EV-cells and p84-cells. A typical Western blot out of three is shown.

To investigate the early molecular events induced by p84 over-expression, the majority of our investigations were performed at 24 h after transfection. In order to explore the mode of cell death, which was preliminarily indicated by cell detachment, we measured the release of the intracellular LDH enzyme in culture medium. As shown in Fig. 2A, the remarkable increase of released LDH clearly indicates that a damage at plasmamembrane occurs. Along with such necrotic-like cell death, an apoptotic mode of cell demise also occurs, as shown by the occurrence of typical apoptotic hallmarks: the significant amount of nuclei showing an apoptotic morphology (Fig. 2B and S1 Fig.), the significant activation of the effector caspases-3/7 (Fig. 2C), and the caspases-3/7-mediated proteolytic cleavage of poly(ADP-ribose) polymerase (PARP) (Fig. 2D).

### Calpain-3 over-expression induces p53 activation and redox imbalance in A375 cells

To give insights into the mechanism responsible for apoptotic cell death in p84-cells, we measured the expression level of a central regulator of susceptibility to apoptosis, i.e. the tumor suppressor protein p53, whose levels are regulated by several stress conditions, including DNA damage, hypoxia, and changes in the redox potential [16]. As shown in Fig. 2E, p53 protein significantly accumulates in p84-cells compared to EV-cells, and such an increase, which is higher at 24 h, mirrors the expression of p84. Along with the stabilization of p53 protein, which does not result from higher mRNA levels (RT-PCR data not shown), its transcriptional activity is also increased, as demonstrated by the expression level of a major p53 target gene, *WAF1/CIP1*, which is 1.7-fold ± 0.1 (mean ± S.E., n = 4, p<0.05) in p84-cells over EV-cells.

**Fig 3:**
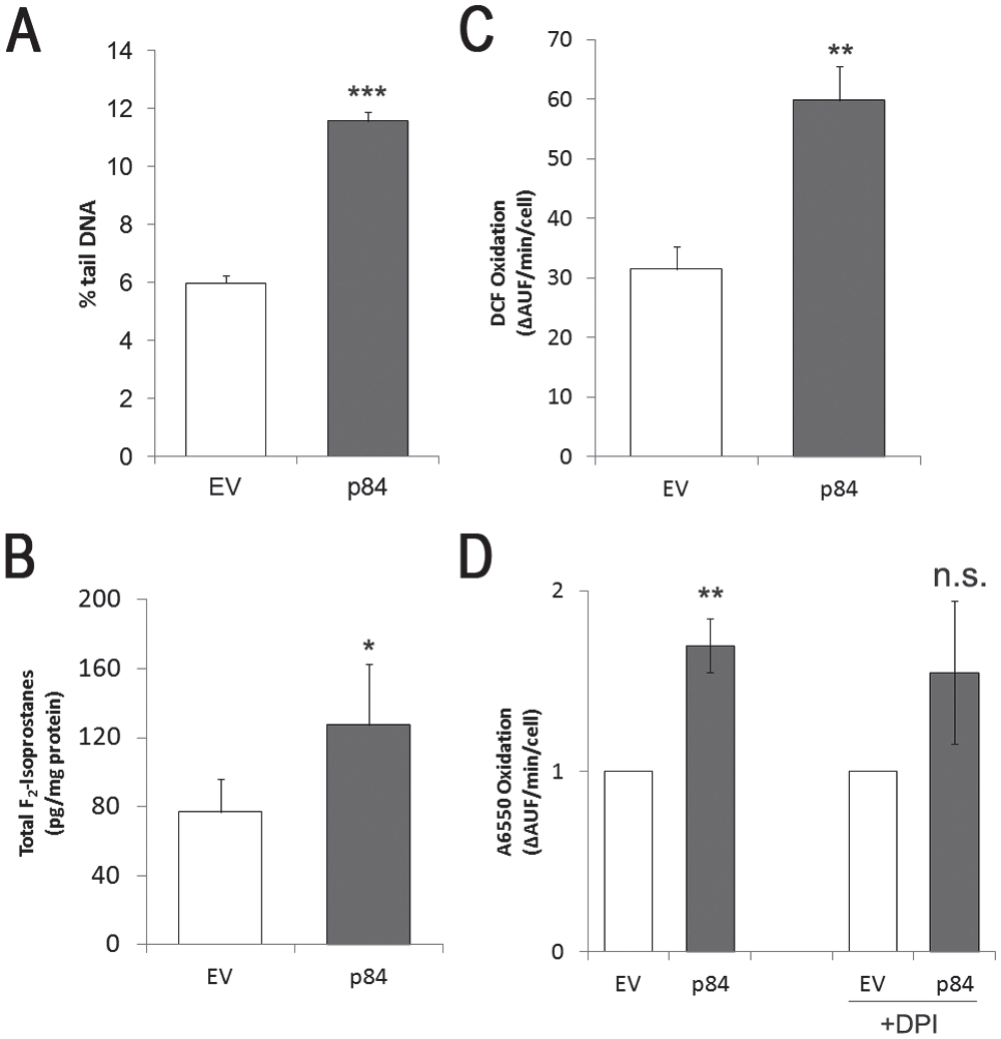
hMp84 induces DNA damage and oxidative alterations of phospholipids in A375 melanoma cells, by stimulating the formation of Reactive Oxygen Species (ROS). In EV-cells and in p84-cells at 24 h after transfection, DNA damage (by means of Comet assay) (**A**) and total (esterified *plus* non-esterified) F_2_-isoprostanes were measured (**B**). In intact EV-cells and p84-cells, the formation of ROS was measured in the intracellular milieu (by DCF-based assay) (**C**), or in the extracellular milieu (by A6550-based assay) with or without the NADPH-oxidase inhibitor, DPI (10 μM), added in the assay mixture (**D**). Results are presented as mean ± S.E. of two independent experiments performed in duplicate (DNA comets), four independent experiments (F_2_-isoprostanes), or six independent experiments (ROS formation). Results in panel D are presented as mean ratio (over EV-cells) ± 95% confidence interval of three independent experiments. *P<0.05, **P<0.01, and ***P<0.001, compared to control EV-cells. n.s.: non-significant, compared to p84-cells without DPI.

In order to evaluate the early events occurring in p84-cells, the biomolecular parameters reported below were measured in adhering cells, where viability is still maintained. Among the stress signals that can lead to p53 stabilization and activation, DNA damage is believed to play a central role. To verify this instance, we measured the basal level of DNA damage by alkaline Comet assay. Higher levels of DNA damage are observed in p84-cells as compared to EV-cells (Fig. 3A), along with an increased level of “hedgehog comets” (S2 Fig.), the latter possibly representing a non-specific indicator of the earliest stages of apoptosis [17,18]. While the significance of hedgehog comets in apoptosis is still debated [18], it is however well established that a Comet positivity is a reliable and sensitive measure of DNA damage, also induced by oxidative stress.

Polyunsaturated fatty acids in membranes lipids can be also modified when a redox imbalance occurs, and lipid peroxidation is a major consequence of oxidative damage, virtually in all cell type. A series of prostaglandin F_2_-like compounds, named F_2_-isoprostanes (F_2_-IsoPs), are formed by free radical-catalyzed, non-enzymatic peroxidation of esterified arachidonic acid in phospholipids. F_2_-IsoPs are chemically stable compounds that are measurable in an accurate and reproducible way, down to the order of picograms; therefore, they are considered specific, sensitive and reliable markers of oxidative stress/lipid peroxidation [19]. A significant amount of total F_2_-IsoPs (esterified *plus* non-esterified) is formed in p84-cells, as compared to EV-cells (Fig. 3B), indicating that cellular membranes (plasmamembrane and/or organelle membranes) undergo lipid peroxidation. In particular, the oxidative alteration of plasmamembrane, which leads to loss of plasmamembrane integrity, could account for the leakage of the intracellular LDH enzyme, as above shown.

In order to directly evaluate whether p84-cells experience an oxidative stress, we measured the intracellular formation of Reactive Oxygen Species (ROS) in intact adhering cells by using the DCF-based assay. A significant amount of oxidant species is formed in p84-cells as compared to EV-cells, at 24 h after transfection (Fig. 3C). In addition to intracellular sources of ROS, which include mitochondrial electron transport chain, cytochromes and, specifically for melanoma cells, melanin metabolism, NADPH oxidase is also considered an important enzyme for extracellular ROS production in melanoma cells [20]. Thus, we investigated whether NADPH oxidase contributes to p84-mediated ROS formation, by using the horseradish peroxidase-linked Amplex Red assay, which measures oxidant species outside the cells. As shown in Fig. 3D, an increase of extracellular peroxides is observed in p84-cells, as compared to EV-cells. However, the NADPH oxidase inhibitor, DPI, added in the *in vitro* assay, does not inhibit such ROS formation, suggesting that NADPH oxidase (at least that working at plasmamembrane level) is not responsible for such redox imbalance, and the measured extracellular ROS are most likely delivered by the intracellular milieu.

To gain further insights into the molecular events underlying the observed oxidative imbalance, in adhering cells at 24 h after transfection we analyzed the expression level of diverse oxidative stress-related genes, by using RT^2^ Profiler PCR Array: Human Oxidative Stress. Out of 84 genes evaluated in the array, 13 genes show expression changes by at least 1.5-fold or 0.5-fold in p84-cells over control EV-cells. For three selected genes showing the highest gene expression changes, Real Time-PCR analysis was performed in additional independent experiments in order to confirm the results obtained by microarray analysis (Table 1). The overall trend of gene expression changes confirms that p84-cells experience an oxidative imbalance. This is consistent with the positive modulation of two genes coding for proteins involved in the production of ROS: *NCF2*, coding for Neutrophilic Cytosolic Factor-2 (p67-Phox), which is a cytosolic activating component of the NADPH oxidase-2 (NOX2) complex, and *NOX4*, coding for the catalytic subunit of NADPH oxidase-4. A different set of genes coding for proteins endowed with various antioxidant properties are also positively regulated, including *GPX2* and *GPX3* coding for Glutathione Peroxidase-2 and-3, *SELS* coding for Selenoprotein S, *GCLM* coding for the modifier subunit of the Glutamate-cysteine ligase (the limiting first enzyme for Glutathione (GSH) biosynthesis), and *SOD2* coding for the mitochondrial Superoxide Dismutase. SOD enzyme, since detoxifies superoxide anion but converts the latter in another oxidant specie (i.e. hydrogen peroxide), can be also regarded as involved in ROS metabolism. Among the antioxidants, it is also observed a significant down-regulation of *GSTZ1* (coding for Glutathione Transferase Z-1), whose deficiency has been proved to induce an oxidative stress and a compensatory up-regulation of *GCLM* [21]. Moreover, a few other genes considered oxidative stress-responsive genes (Table 1) are also up-regulated in p84-cells.

**Table 1:** Gene expression analysis of oxidative stress-related genes in A375 melanoma cells over-expressing hMp84.

Gene symbol (accession number)	Description	Gene name	Fold-change
Genes involved in ROS Production/Metabolism			
**NCF2** (NM_000433)	Neutrophil cytosolic factor 2	P67-PHOX	2.54 ± 0.32 p<0.01
**NOX4** (NM_016931)	NADPH oxidase 4	RENOX	1.7
**SOD2** (NM_000636)	Superoxide dismutase 2, mitochondrial	MNSOD	1.6
Oxidative Stress-Responsive Genes, encoding Antioxidants			
**GPX2** (NM_002083)	Glutathione peroxidase 2	GSHPx-2	1.9
**GPX3** (NM_002084)	Glutathione peroxidase 3	GSHPx-3	1.5
**SELS** (NM_203472)	Selenoprotein S	ADO15	1.5
**GCLM** (NM_002061)	Glutamate-cysteine ligase, modifier subunit	GLCLR	1.6
**GSTZ1** (NM_001513)	Glutathione transferase zeta 1	GSTZ1–1	0.44 ± 0.05 p<0.01
* **SOD2** *(NM_000636)*	Superoxide dismutase 2, mitochondrial	MNSOD	1.6
Oxidative Stress Responsive Genes			
**CCL5** (NM_002985)	Chemokine (C-C motif) ligand 5	RANTES	2.75 ± 0.45 p<0.05
**DUSP1** (NM_004417)	Dual specificity phosphatase 1	MKP-1	1.7
**FTH1** (NM_002032)	Ferritin, heavy polypeptide 1	PIG15	1.5
**HMOX1** (NM_002133)	Heme oxygenase 1	HO-1	1.5
**PTGS2** (NM_000963)	Prostaglandin G/H synthase and cyclooxygenase	COX-2	1.5

Gene expression was analyzed in triplicate samples by using RT^2^ Profiler PCR Array—Human Oxidative Stress. For *NCF2*, *GSTZ1* and *CCL5*, three additional independent experiments in triplicates were performed by RT-PCR. Gene expression values in p84-cells are expressed as fold-change (mean ± S.E.) over EV-cells after 24 h of transfection.

*SOD2 has been included both in genes involved in Reactive Oxygen Species (ROS) production/metabolism and in oxidative stress-responsive genes, encoding antioxidants.

### Calpain-3-mediated cell injury in A375 cells is prevented by NAC and pifithrin-α

**Fig 4:**
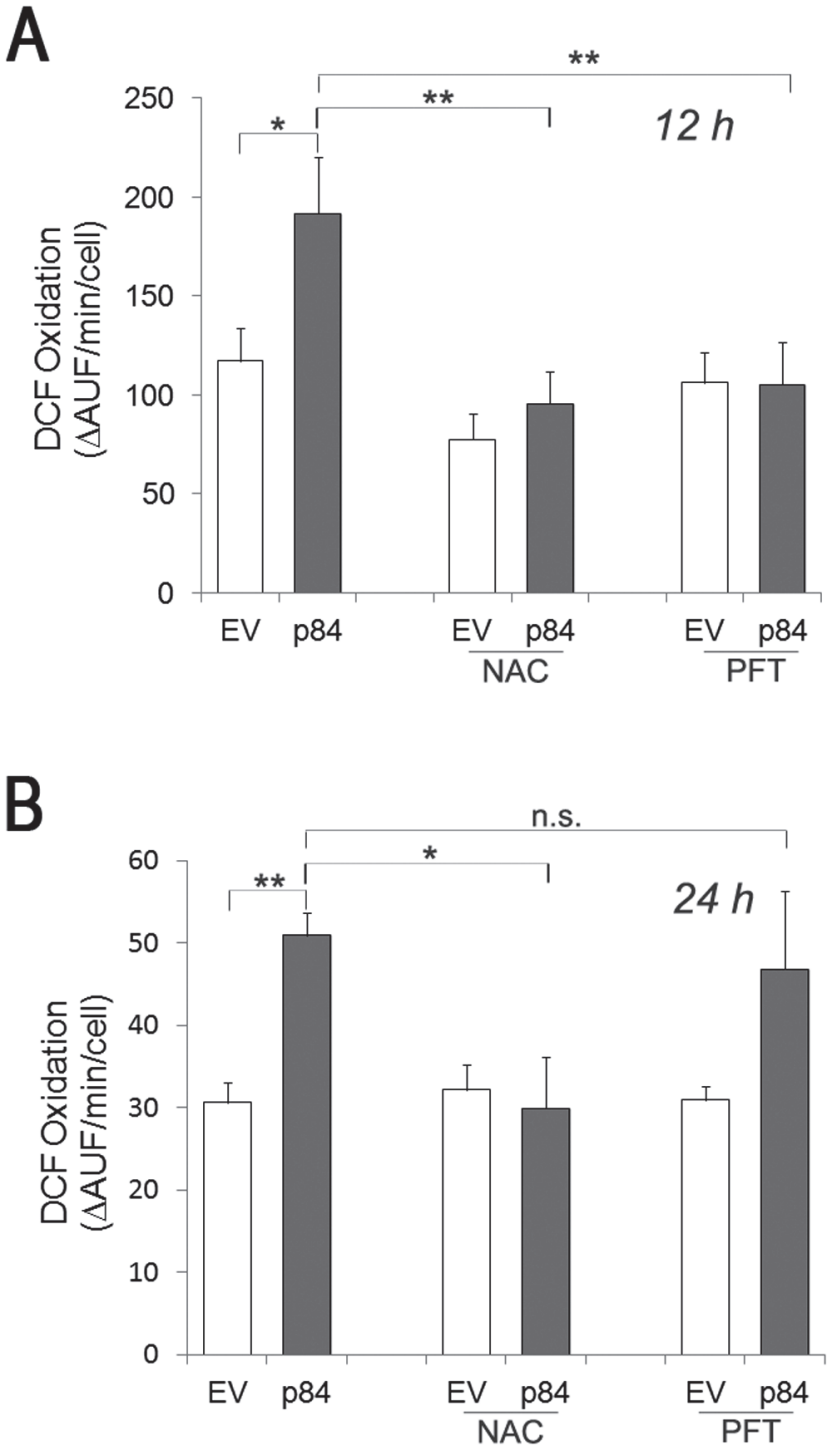
Treatment with the antioxidant *N*-Acetyl-L-Cysteine (NAC) or with the p53 inhibitor, Pifithrin-α (PFT), prevents ROS formation in hMp84-overexpressing A375 cells. At 12 h (**A**) and 24 h (**B**) after transfection, ROS formation was evaluated by DCF-based assay in EV-cells and p84 cells, incubated with or without NAC (5 mM) or PFT (1 μM). Results are presented as mean ± S.E. of six (A) or three (B) independent experiments. *P<0.05; **P<0.01; n.s.: non-significant.

**Fig 5:**
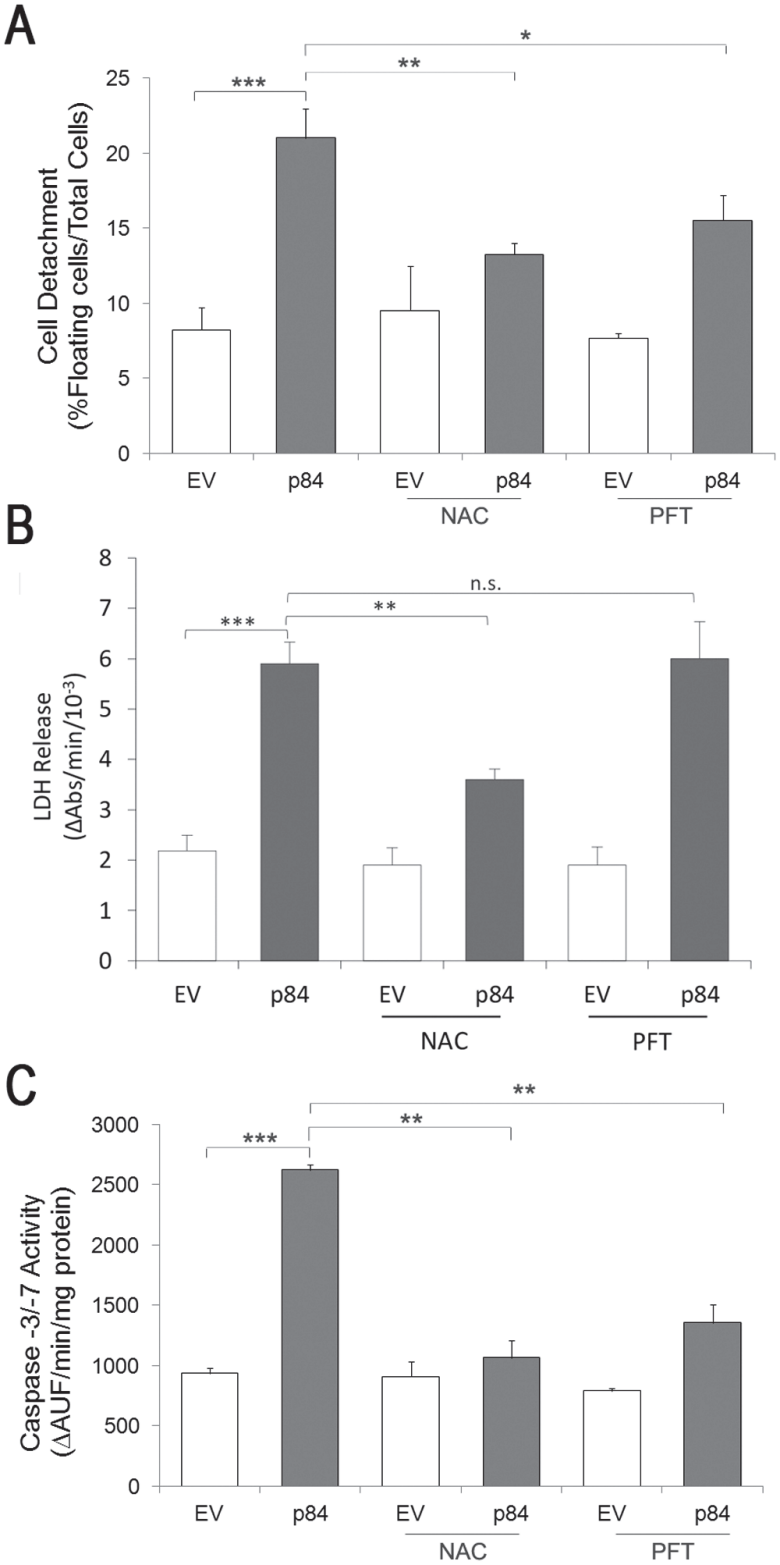
Treatment with the antioxidant *N*-Acetyl-L-Cysteine (NAC) or with the p53 inhibitor, Pifithrin-α (PFT), prevents cell death in hMp84-overexpressing A375 cells. At 24 h after transfection, in EV- and p84-cells, incubated with or without NAC (5 mM) or PFT (1 μM), cell death was evaluated as percentage of floating cells on total cells (**A**), as LDH released in the culture medium (**B**), and as caspase-3/-7 enzymatic activity (**C**). Results are presented as mean ± S.E. of three (A and B) or five (C) independent experiments. *P<0.05, **P<0.01, ***P<0.001; n.s.: non-significant.

To understand whether the redox imbalance occurring in p84-cells has a causative role in cell proliferation impairment and in cell injury, we treated EV- and p84-cells with the antioxidant N-acetyl-cysteine (NAC) [22]. While efficiently preventing the formation of intracellular ROS in p84-cells at 12 h (Fig. 4A) and 24 h (Fig. 4B) after transfection, NAC treatment significantly recovers viability of p84-cells, as revealed by the lower number of detached cells (Fig. 5A), along with a remarkable decrease of both LDH release (Fig. 5B) and caspase-3/-7 activity (Fig. 5C). These NAC-mediated effects suggest that an antioxidant environment is able to counteract both an apoptotic and necrotic cell damage in p84-cells. On the contrary, no significant restoration of cell proliferation is afforded by NAC (data not shown).

To investigate the role played by p53 stabilization in p84-cells, we treated EV- and p84-cells with pifithrin-α (PFT), a compound which reversibly inhibits the transcriptional activity of p53 [23]. PFT treatment decreases to some extent the hMp84-mediated cell injury, as firstly indicated by the lower percentage of detached cells (Fig. 5A). Such a protection is mainly effective on apoptotic cell death, as shown by the remarkable inhibition of caspase-3/-7 activity (Fig. 5C); on the contrary, no protective effect is evident on plasmamembrane damage (Fig. 5B). As concerns the pro-oxidant status in p84-cells, p53 inhibition by PFT completely abrogates ROS formation at early times (12 h) (Fig. 4A) after transfection, but such effect is lost at 24 h (Fig. 4B), possibly due to the reversible, and hence transient, inhibition of p53 by PFT. Similarly to NAC treatment, PFT treatment is not able to recover cell proliferation (data not shown).

### Calpain-3 over-expression affects cell proliferation, and stimulates cell death and oxidant species formation in HT-144 melanoma cells

**Fig 6:**
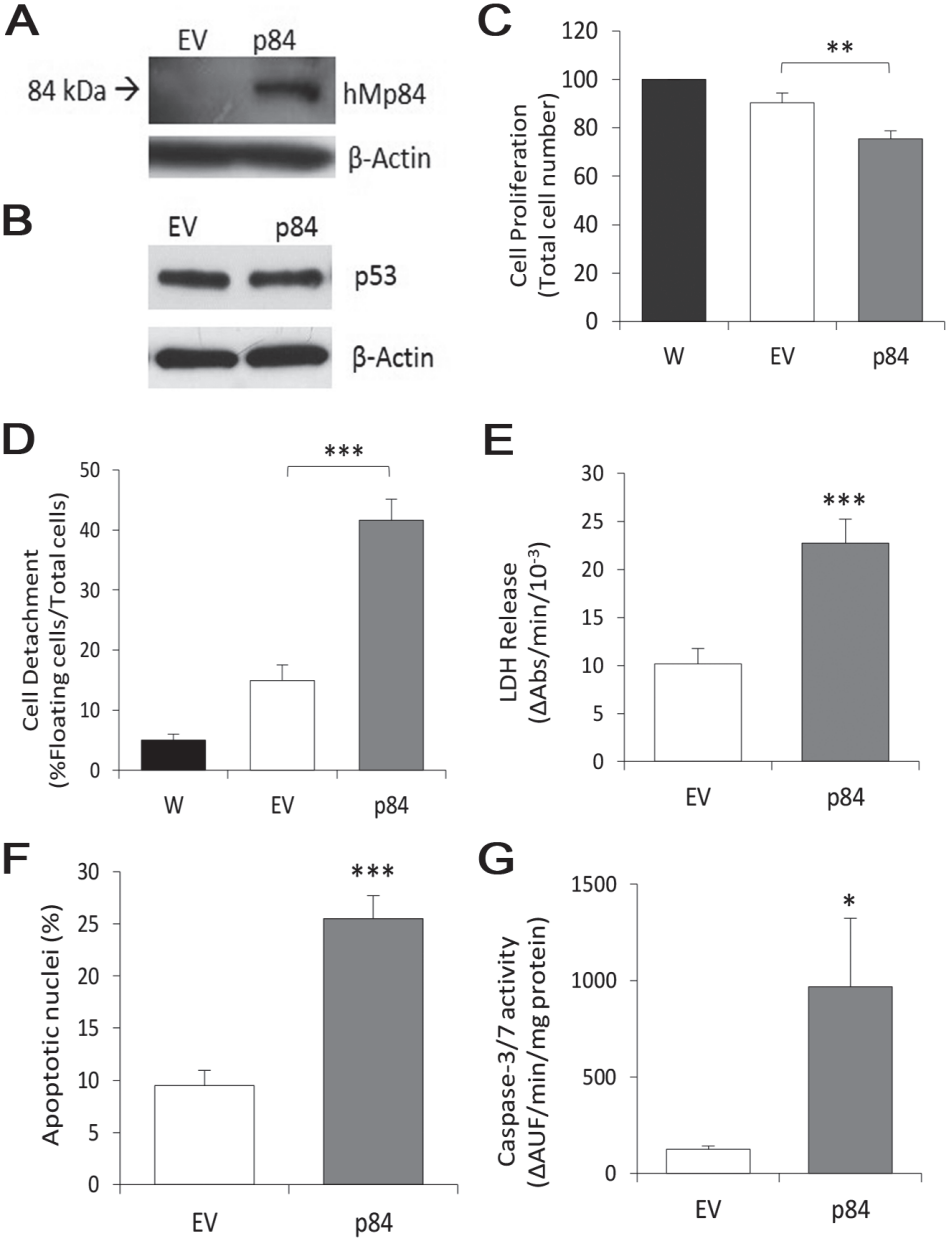
Transient over-expression of hMp84 impairs cell proliferation and stimulates cell death in HT-144 melanoma cells. All parameters were evaluated at 24 h after transfection. (**A**) hMp84 protein (p84) was evaluated by Western blot in adhering cells of empty vector- (EV) and hMp84-transfected cells (p84); a typical Western blot out of three is shown. (**B**) p53 protein was evaluated by Western blot; a typical experiment out of four is shown. (**C**) Cell proliferation (total cell number) and (**D**) cell detachment (% of floating cells on total cells) were evaluated in parental, non-transfected cells (W), in EV- and in p84-cells. Cell death was evaluated as amount of intracellular LDH released in the culture medium (**E**), as percentage of nuclei showing an apoptotic morphology (Hoechst 33258 staining) (**F**), and as caspase-3/-7 enzymatic activity (**G**). Results are presented as mean ± S.E. of at least five independent experiments. *P<0.05; **P<0.01; ***P<0.001.

The major findings obtained in A375 cells were confirmed in HT-144 melanoma cells over-expressing hMp84 (p84-cells). As shown in Fig. 6A, in empty vector-transfected cells (EV-cells) p84 is hardly detectable, while in p84-cells there is a notable amount of the protein at 24 h after transfection. As expected, a huge increase of hMp84 mRNA is also observed in p84-cells (RT-PCR data not shown), compared to non-transfected cells and EV-cells. Similarly to A375 cells, at 24 h after transfection, Calpain-3 over-expression lowers cell proliferation, evaluated as total cell number (Fig. 6C), compared to EV-cells. A remarkable increase of floating cells detached from the monolayer is also observed, as a first indication of ongoing cell damage and eventual cell demise (Fig. 6D). In HT-144 p84-cells, cell death occurs indeed, as documented by the remarkable release of intracellular LDH in culture medium (Fig. 6E), by the significant amount of nuclei showing an apoptotic morphology (Fig. 6F and S3 Fig.), and by the significant activation of caspases-3/-7 (Fig. 6G). Differently from A375 cells, no accumulation of p53 protein in observed at 24 h after transfection (Fig. 6B).

**Fig 7:**
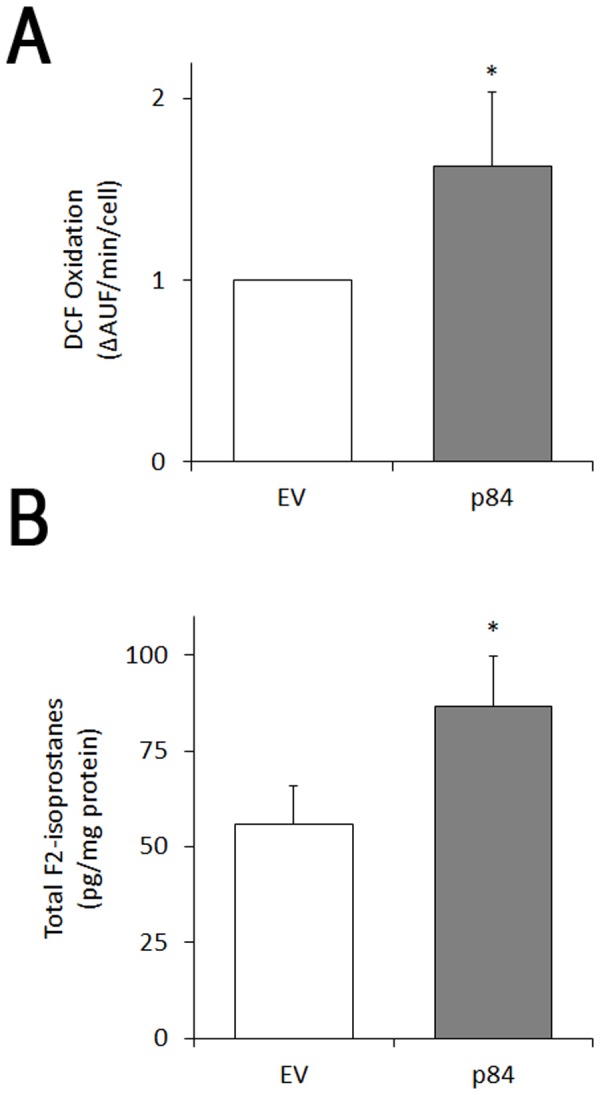
hMp84 stimulates the formation of Reactive Oxygen Species (ROS) and induces oxidative alterations of phospholipids in HT-144 melanoma cells. At 24 h after transfection, the formation of intracellular ROS (by DCF-based assay) (**A**) and total (esterified *plus* non-esterified) F_2_-isoprostanes (**B**) were measured in adhering EV- and p84-cells. Results are presented as mean ± S.E. of three to five independent experiments. *P<0.05.

hMp84 over-expression also alters the redox state in HT-144 cells, as shown by the increased level of intracellular Reactive Oxygen Species (revealed by the DCF-based assay) formed in p84-cells compared to EV-cells (Fig. 7A). Such redox imbalance leads to lipid peroxidation of plasmamembrane and/or organelle membranes, as revealed by the increased formation of F_2_-isoprostanes in p84-cells (Fig. 7B).

### Over-expression of mutant Calpain-3 (hMp84^C42S^) does not affect cell fate in both A375 and HT-144 melanoma cells

**Fig 8:**
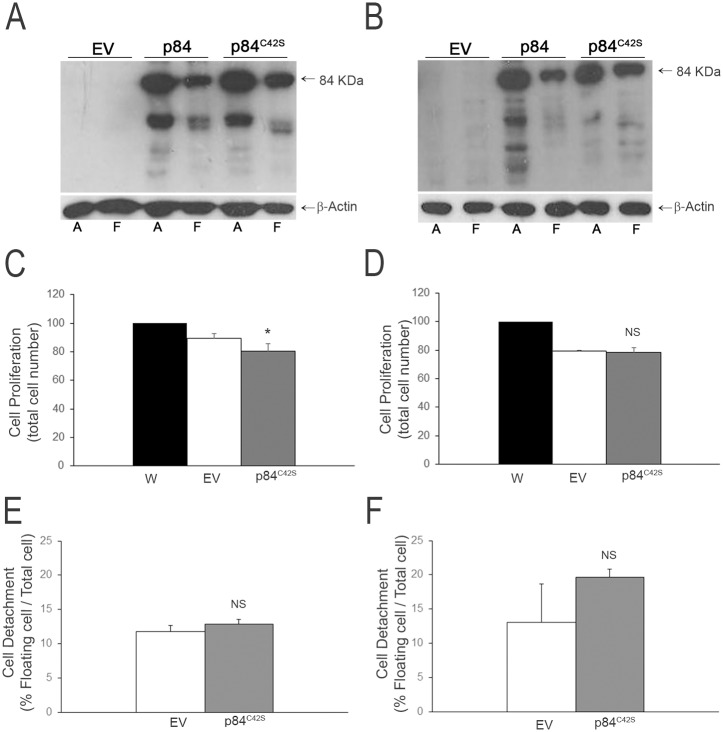
Over-expression of hMp84 mutated in the active site (hMp84^C42S^) does not affect cell fate in both A375 and HT-144 melanoma cells. All parameters were evaluated at 24 h after transfection. hMp84^C42S^ protein was evaluated by Western blot in adhering and floating cells of A375- (**A**) and HT-144-transfected cells (**B**); typical Western blots out of three are shown. Cell proliferation (total cell number) was evaluated in parental, non-transfected cells (W), in EV- and in hMp84^C42S^-cells of A375 (**C**) and HT-144 cells (**D**). Cell detachment was evaluated in EV- and in hMp84^C42S^-cells of A375 (**E**) and HT-144 cells (**F**). Results are presented as mean ± S.E. of at least three independent experiments. NS = non-significant and *P = 0.046 compared to EV-cells.

In order to understand whether the observed cellular events required a potentially active Calpain-3, in selected experiments we overexpressed the mutant hMp84^C42S^ (virtually devoid of its enzymatic activity) in both melanoma cell lines. First of all, in hMp84^C42S^–overexpressing cells (hereafter called p84m-cells) the amount of full-length hMp84^C42S^ is comparable to the overexpressed full-length hMp84. The pattern of fragments at lower molecular weight is also comparable, although it is quantitatively lighter in HT-144 p84m-cells (Fig. 8A and B). The similarity of the proteolysis pattern between hMp84 and hMp84^C42S^ suggests that autolysis of this Calpain-3 variant is not relevant as for muscle Calpain-3, and other proteases are likely responsible for the observed processing. As concerns the biological effects of the mutant variant, at 24 h after transfection, with the exception of a minor decrease of the total cell number in A375 p84m-cells (p = 0.046), p84m-cells do not show neither increase of cell detachment (which is a first sign of cell damage and eventual cell death) nor significant decrease of total cell number, compared to EV-cells (Fig. 8C-F). These results suggest that a potentially active hMp84 is required for its detrimental effects in melanoma cells.

## Discussion

Calpain-3 is a Ca^2+^-regulated cysteine protease, predominantly expressed in skeletal muscle, where it is essential to maintain healthy conditions of this tissue. The importance of its activity has been clearly assessed in human muscles, where several described missense mutations in the gene, giving a protease devoid of its proteolytic activity, are responsible for Limb Girdle Muscular Dystrophy type 2A (LGMD2A), an autosomal recessive disease characterized by progressive atrophy and weakness of the proximal limb muscles [2,24]. Muscle Calpain-3 undergoes a rapid autoproteolysis (both *in vivo* and *in vitro)*, which leads to its enzymatic activation [25]. Its relative instability makes difficult the identification of physiological substrates other than itself. However, in various experimental settings (mainly *in vitro* assays), diverse cleavage substrates have been suggested [3,15,26–31], a few of which shared with conventional calpains.

Several variants of Calpain-3 have been reported in many normal mammalian cells [32], including rodent ocular tissues [33,34] and astrocytes [35], and human lymphocytes [36], thus suggesting that Calpain-3 is also important for tissues other than skeletal muscle. As concerns tumor cells, a very short variant 6 has been identified in melanoma cells [32]; this variant is down-regulated in drug-induced terminal differentiation of melanoma cells, but its over-expression does not significantly affect growth kinetics and cell viability [8]. A previous study of our group has identified two novel splicing variants of *CAPN3*, namely hMp78 and hMp84, in human melanoma cell lines and melanocytic lesions [4], both variants displaying an atypical initiation exon, instead of the canonical NS-encoding exon 1, and the shorter variant lacking the IS1-coding exon 6. On the basis of this previous work, which also suggested an active role of these isoforms in the apoptotic machinery of melanoma cells, in the present study we have further investigated the biological role of the longer variant hMp84 in this tumor cell type.

In A375 and in HT-144 melanoma cells over-expressing hMp84 (p84-cells), we observe a variety of significant changes compared to control EV-cells. It is worthwhile to highlight that the transfection efficiency obtained by us is partial (around 40 and 30%, in A375 and HT-144 cells, respectively), thus all observed changes, as measured in the whole cell population, are even underestimated. First of all, a significant production of Reactive Oxygen Species (ROS) occurs. Several intracellular sources of ROS have been proposed in melanoma cells [37–39], such as the mitochondrial electron transport chain [40], considered the major source in metabolically very active tumor cells, NADPH oxidases (mainly Nox4) [41,42] and, as unique feature of these melanocytes-derived tumor cells, the aberrant melanin biosynthesis and oxidation [43]. Among these, we have not strictly ascertained which is the source of ROS in p84-cells; however, the results obtained in A375 cells suggest that the major production of ROS derives from the intracellular milieu. In this respect, the significantly increased gene expression of *NCF2*, coding for the cytosolic activating component p67-Phox of the plasmamembrane NOX2 complex, could potentially contribute to intracellular ROS generation, in case that in melanoma cells NOX2 is located also into intracellular membranes, as demonstrated in different cell types [41].

As a compensatory attempt to protect themselves against a ROS-mediated cell injury, A375 p84-cells tend to up-regulate, albeit mildly, several genes encoding for antioxidant enzymes. In this antioxidant context, the remarkable down-regulation of *GSTZ1* needs to be valued from a different perspective. *GSTZ1* codes for a member of Glutathione-S-transferases, which catalyze the conjugation of glutathione (GSH) to a variety of electrophilic substances [44], including oxidative stress-generated products, and also modulate cell signaling pathways that control cell proliferation and apoptosis [45]. Interestingly, a deficiency of GSTZ1 has been demonstrated to cause oxidative stress and activation of antioxidant pathways, including the modifier subunit of glutamate-cysteine ligase, the limiting enzyme for glutathione biosynthesis [21]. All these mechanisms are expected to be operative also in p84-cells, where a significant down-regulation of *GSTZ1* occurs.

Over the past few years, many evidences have highlighted the subtle balance existing between p53 and redox homeostasis: on one hand, ROS can oxidize cysteine residues of p53, modifying its conformational status and transcriptional activity; on the other hand, p53 can influence cellular ROS production, by activating or repressing many ROS-regulating genes coding for pro-oxidant or anti-oxidant proteins [46]. In our study, Calpain-3-overexpressing A375 cells show both a significant accumulation of p53 (wild-type in these cells) and expression changes of several oxidative stress-related genes which are also p53-target genes (such as *NCF2*, *SOD2*, *GPX2*, *GPX3*) [46–49], suggesting that p53 plays a role in the complex network of ROS regulation. Since ROS are actually formed, at levels sufficient to induce oxidative modification of both DNA and phospholipids, we can argue that the contribution of p53 in the redox homeostasis results in a net shift of the redox balance toward a pro-oxidant status. This is also confirmed by the prevention of ROS formation afforded by PFT-α, an inhibitor of p53 transcriptional activity. However, a p53-independent mechanism for ROS formation must be also considered. In Calpain-3-overexpressing HT-144 cells, in fact, the increased formation of ROS is not accompanied by up-regulation of p53 protein (wild-type also in these cells). This is likely due to homozygous mutation in ATM (Ataxia Telangiectasia Mutated) gene [50], that is an important player in p53 activation. Interestingly, the fact that no increase of p53 transcriptional activity is expected to occur in HT-144 p84-cells extends the biological role of Calpain-3 to those tumor types (including melanomas to some extent) harbouring *TP53* mutations which impair p53 transcriptional activity.

It is well known that ROS, acting as second messengers, modulate multiple cell signalling pathways, and such pleiotropic effect can result in opposite cell fates [51]. Redox alterations due to elevated ROS produced by different physiological sources have been proved to be beneficial for diverse tumor types, including melanoma [37,39]. By favouring tumor cell proliferation [20] and genomic instability [52], they contribute to cancer progression. On the other hand, it is also well established (more than one hundred articles in PubMed) that an excessive or lasting ROS production can culminate in apoptotic death of melanoma cells. Here, we show that melanoma cells with enforced expression of Calpain-3 experience a redox imbalance and, judging on their adverse cell fate (i.e. stimulation of cell death), we can say that they experience an oxidative stress. The causative role of such oxidative stress in the loss of cell viability is substantiated, first, by the significant protection against cell death afforded by the antioxidant NAC. Second, the causative role of p53-dependent oxidative stress is also indicated by the partial rescue occurring in PFT-α-treated cells; in these p53-inhibited cells, however, additional anti-apoptotic mechanisms can be also evoked [16].

While efficiently preventing ROS formation and cell death, the antioxidant NAC is not able to restore the proliferation of Calpain-3-overexpressing cells, suggesting that ROS do not play a major role in impairing cell growth in this experimental context. Rather, Calpain-3 could directly effect a degradative processing or a regulatory modification (through limited proteolysis) of target proteins involved in cell cycle control. Consistently, in different cells over-expressing the muscle-specific Calpain-3, a number of proteolyzed substrates have been identified, including components of the protein synthesis apparatus, glycolytic enzymes [26], and β-catenin [29]. Interestingly, β-catenin, a key component of Wnt pathway proved to be often up-regulated in melanomas [53], co-activates transcription factors involved in cell proliferation, survival and migration. In this context, the proteolytic activity of hMp84 could negatively control the basal or up-regulated level of free β-catenin.

A further event occurring in A375 cells is the significantly increased expression of *RANTES*, an oxidative stress—responsive gene coding for CCL5 chemokine. It has been reported that CCL5 is secreted by melanoma cells *in vivo* and *in vitro*, and is able to recruit diverse tumor-infiltrating leucocytes (TIL) [54,55]. Interestingly, in various experimental settings (in *in vitro* treated melanoma cells, in melanoma-bearing mice, and in melanoma patients), an overall antitumor effect of this cytokine has been demonstrated [54]. These findings are consistent with our results, where up-regulation of *RANTES* in Calpain-3-overexpressing cells could contribute to the impairment of cell growth and to cell death.

As concerns the catalytic *versus* structural role of Calpain-3, it was demonstrated that knock-in mice which express a structurally intact but protease-inactive Calpain-3 (C129S mutant) have a dystrophic muscle phenotype less severe than *CAPN3* ^−/−^ mice, thus proving that muscle Calpain-3 also has a structural, non-proteolytic function [56]. Differently, in our experimental setting the absence of macroscopic signs of cell damage in cells overexpressing hMp84^C42S^ (virtually devoid of enzymatic activity) allows us to conclude that hMp84 catalytic activity is required for the observed detrimental effects in melanoma cells.

In conclusion, in the present study we show that in melanoma cells high levels of virtually active Calpain-3 (variant hMp84) stimulate a variety of mechanisms which are eventually detrimental for cells, including p53 accumulation, modulation of several oxidative stress-related genes, increased formation of ROS (Reactive Oxygen Species) leading to oxidative modification of phospholipids, and DNA damage. A potential role of Calpain-3 in melanoma progression can be envisaged, consistently with our previous observations showing lower expression level (compared to benign lesions) of both Calpain-3 variants, hMp78 and hMp84, in vertical growth phase melanomas and, even lower, in metastases [4]. These most aggressive phenotypes, in fact, are expected to benefit from down-regulation of mechanisms which impair cell growth and stimulate cell death.

## Supporting Information

S1 Fig hMp84 induces an apoptotic nuclear morphology in A375 melanoma cells. Representative fluorescence microscope images of Hoechst 33342-stained nuclei at 24 h after transfection; 10x (left panels) and 40x (right panels) magnification. Empty vector-transfected cells (EV-cells) and hMp84-transfected cell (p84-cells). Arrows indicate condensed and fragmented nuclei. Scale bar = 50 μm.
(TIF)

S2 Fig hMp84 induces DNA damage in A375 melanoma cells. Representative fluorescence microscope images of comets. (**A**) Empty vector-transfected cells (EV-cells); (**B**) hMp84-transfected cell (p84-cells); (**C**) a hedgehog comet in p84-cells. Scale bars = 10 μm.
(TIF)

S3 Fig hMp84 induces an apoptotic nuclear morphology in HT-144 melanoma cells. Representative fluorescence microscope images of Hoechst 33342-stained nuclei at 24 h after transfection; 10x (left panels) and 40x (right panels) magnification. Empty vector-transfected cells (EV-cells) and hMp84-transfected cell (p84-cells). Arrows indicate condensed and fragmented nuclei. Scale bar = 50 μm.
(TIF)

S1 Table Primers used for gene expression analysis by RT-PCR, hMp84 cloning (*) and mutagenesis experiments (**). (DOC)
