# Supplementary figures and images for: Calpain-3 Impairs Cell Proliferation and Stimulates Oxidative Stress-Mediated Cell Death in Melanoma Cells
Source: PLoS One. 2015 Feb 6;10(2):e0117258. doi: 10.1371/journal.pone.0117258 (PMC4319969; doi:10.1371/journal.pone.0117258)

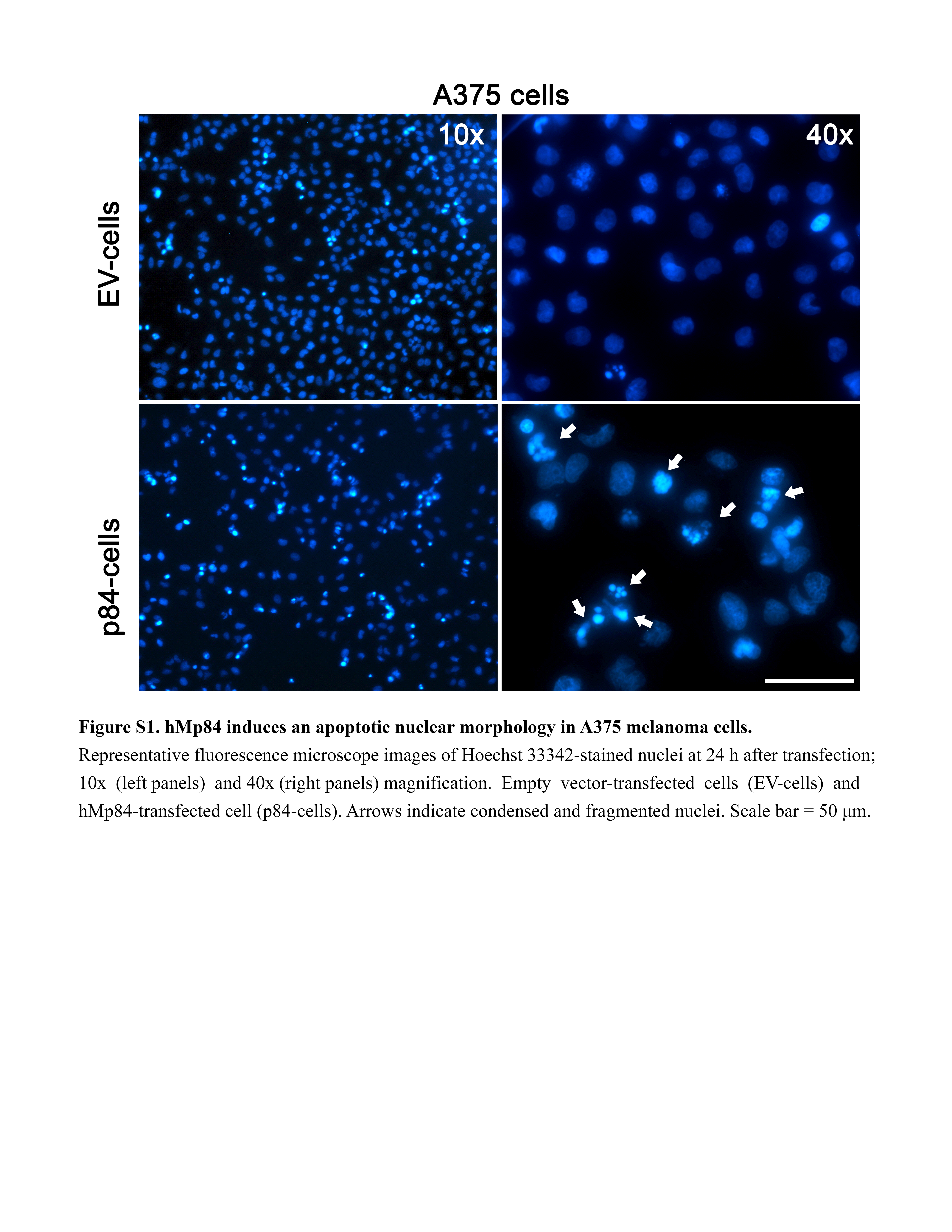

Supplement: S1 Fig — Representative fluorescence microscope images of Hoechst 33342-stained nuclei at 24 h after transfection; 10x (left panels) and 40x (right panels) magnification. Empty vector-transfected cells (EV-cells) and hMp84-transfected cell (p84-cells). Arrows indicate condensed and fragmented nuclei. Scale bar = 50 μm. (TIF) [file pone.0117258.s001.tif]

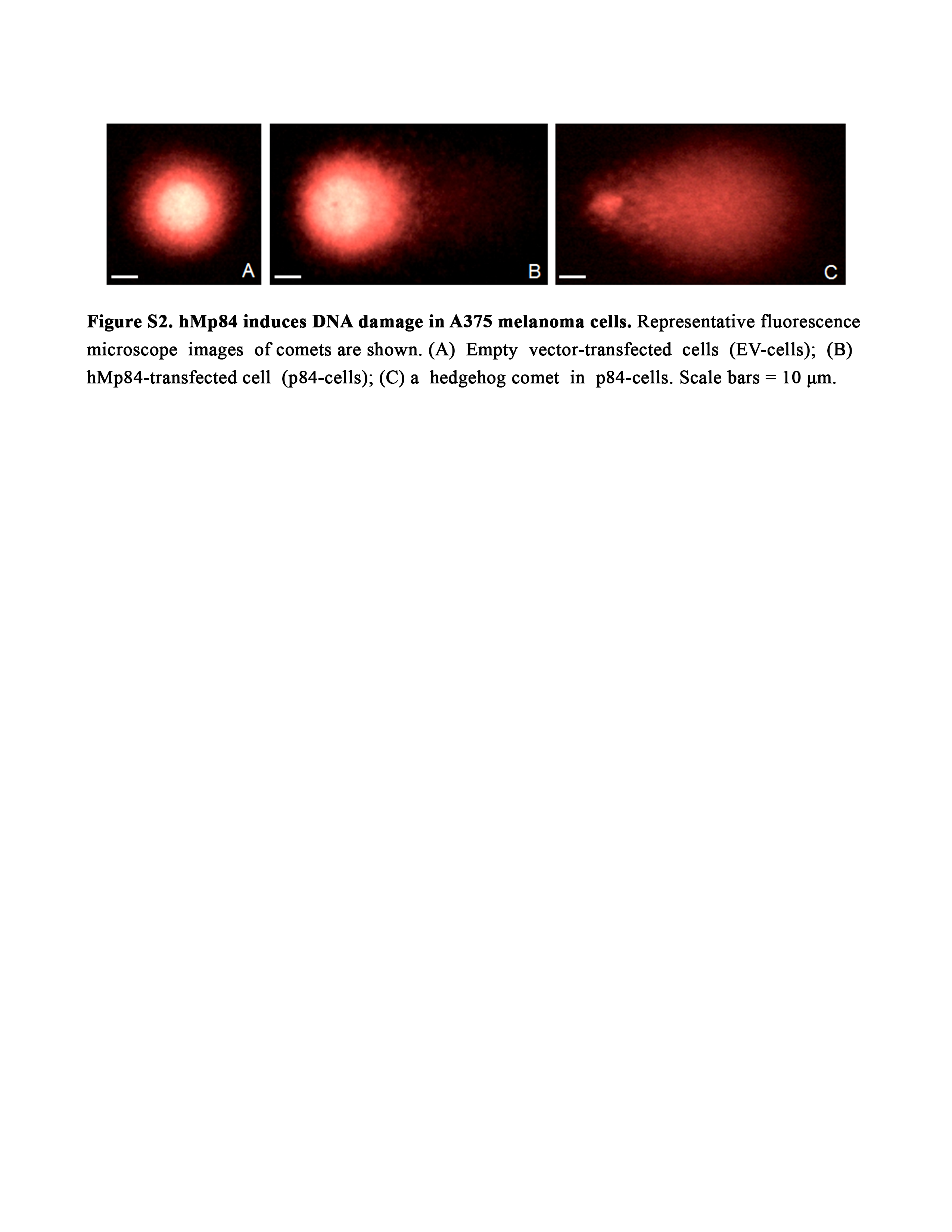

Supplement: S2 Fig — Representative fluorescence microscope images of comets. (A) Empty vector-transfected cells (EV-cells); (B) hMp84-transfected cell (p84-cells); (C) a hedgehog comet in p84-cells. Scale bars = 10 μm. (TIF) [file pone.0117258.s002.tif]

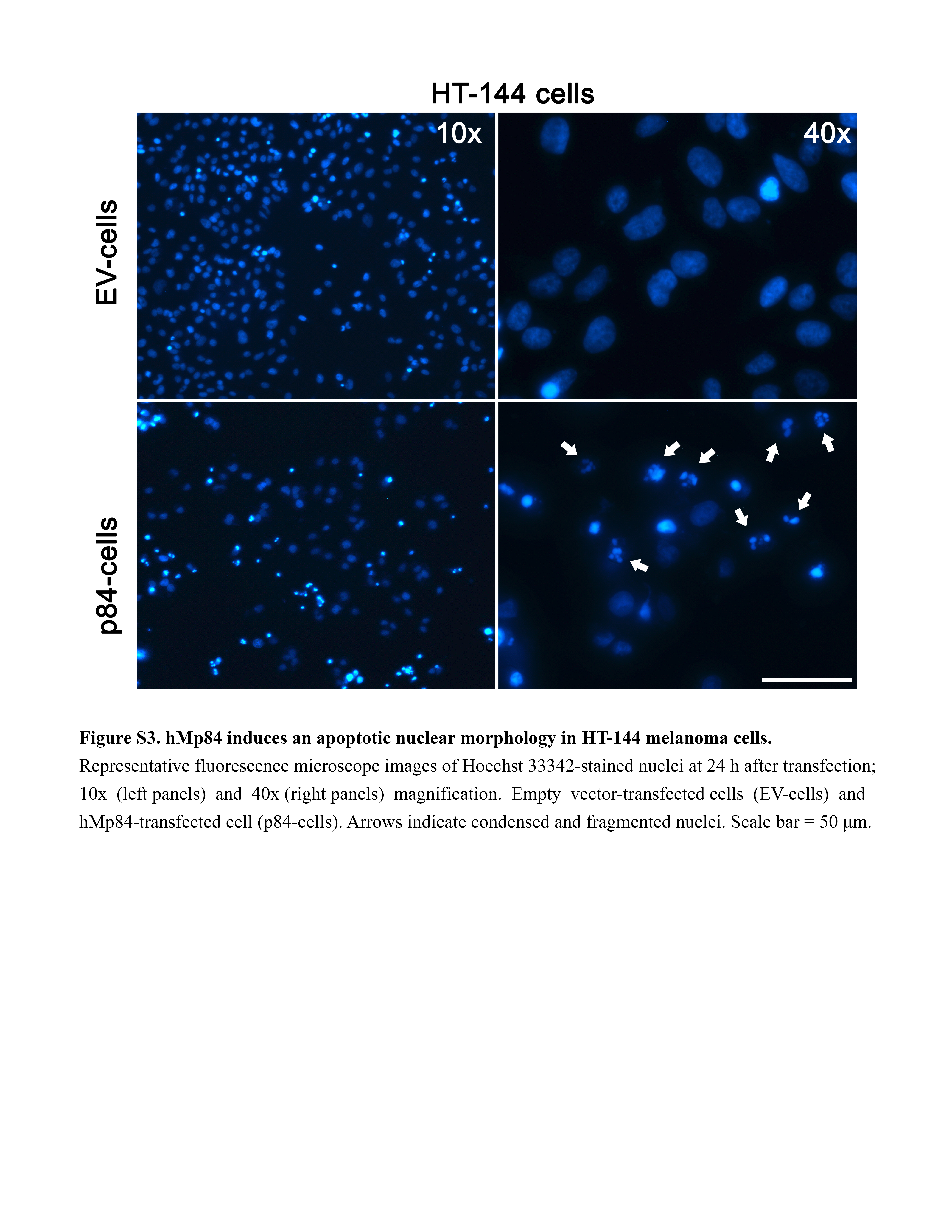

Supplement: S3 Fig — Representative fluorescence microscope images of Hoechst 33342-stained nuclei at 24 h after transfection; 10x (left panels) and 40x (right panels) magnification. Empty vector-transfected cells (EV-cells) and hMp84-transfected cell (p84-cells). Arrows indicate condensed and fragmented nuclei. Scale bar = 50 μm. (TIF) [file pone.0117258.s003.tif]
