# Supplementary material for: Calpain-3 Impairs Cell Proliferation and Stimulates Oxidative Stress-Mediated Cell Death in Melanoma Cells
Source: PLoS One. 2015 Feb 6;10(2):e0117258. doi: 10.1371/journal.pone.0117258 (PMC4319969; doi:10.1371/journal.pone.0117258)
Supplement: S1 Table — (DOC) [file pone.0117258.s004.doc]

**Table S1**

Primers used for gene expression analysis (RT-PCR), hMp84 cloning (*) and site-directed mutagenesis (**).

| **Gene** | **Sequence of primers** |
| --- | --- |
| hMp84 | *For 5’-CGTGGATCGGCCAGTGAAAAA - 3’*  *Rev 5’- CTCTGACTCCTGGTCCAC - 3’* |
| TP53 | *For 5’ - TGGAAGGAAATTTGCGTGTGG - 3’*  *Rev 5’ - TCAGTCTGAGTCAGGCCCTTC - 3’* |
| WAF1/CIP1 | *For 5’- AGTCAGTTCCTTGTGGAGCC - 3’*  *Rev 5’- GCATGGGTTCTGACGGACAT - 3’* |
| NCF2 | *For 5’- TGTCACCAGGCCAGAAACAA - 3’*  *Rev 5’- TGCACCTTGAGTGTGTAGGG - 3’* |
| GSTZ1 | *For 5’- ACTTTCTCCACAAAAGCCCA - 3’*  *Rev 5’- CGTCTCGTAGTCGATGCCTT - 3’* |
| CCL5 | *For 5’ - CTGCTTTGCCTACATTGCCC - 3’*  *Rev 5’ - TCGGGTGACAAAGACGACTG - 3’* |
| ACT | *For 5’ - CCAACCGCGAGAAGATGA - 3’*  *Rev 5’ - CAGCCTGGATAGCAACGT - 3’* |
| * hMp84 | *For 5’- GCGGCGGATCCATGAGTTGGCAAATCAG - 3’*  *Rev 5’- GCGGCCTCGAGTCAGGCATACATGGTGAG - 3'* |
| ** hMp84^C42S^ | *For 5’- GGAGAGCTAGGGGACAGTTGGTTTCTCGCAGCC - 3’*  *Rev 5’- GGCTGCGAGAAACCAACTGTCCCCTAGCTCTCC - 3’* |
